# Characterization and Genomic Analysis of BUCT549, a Novel Bacteriophage Infecting *Vibrio alginolyticus* With Flagella as Receptor

**DOI:** 10.3389/fmicb.2021.668319

**Published:** 2021-06-17

**Authors:** Jing Li, Fengjuan Tian, Yunjia Hu, Wei Lin, Yujie Liu, Feiyang Zhao, Huiying Ren, Qiang Pan, Taoxing Shi, Yigang Tong

**Affiliations:** ^1^College of Life Science and Technology, Beijing University of Chemical Technology, Beijing, China; ^2^Qingdao Phagepharm Bio-tech Co., Ltd., Shandong, China; ^3^Academy of Military Medical Sciences, Beijing, China

**Keywords:** *vibrio alginolyticus*, bacteriophage, receptor, MSHA protein, genome analysis

## Abstract

*Vibrio alginolyticus* is one of the most important of pathogens that can infect humans and a variety of aquatic animals, and it can cause food poisoning and septicemia in humans. Widely used antibiotics are gradually losing their usefulness, and phages are gaining more attention as new antibacterial strategies. To have more potential strategies for controlling pathogenic bacteria, we isolated a novel *V. alginolyticus* phage BUCT549 from seafood market sewage. It was classified as a new member of the family *Siphoviridae* by transmission electron microscopy and a phylogenetic tree. We propose creating a new genus for BUCT549 based on the intergenomic similarities (maximum is 56%) obtained from VIRIDIC calculations. Phage BUCT549 could be used for phage therapy due to its stability in a wide pH (3.0–11.0) range and high-temperature (up to 60°C) environment. It had a latent period of 30–40 min and a burst size of 141 PFU/infected bacterium. In the phylogenetic tree based on a terminase large subunit, BUCT549 was closely related to eight *Vibrio* phages with different species of host. Meanwhile, our experiments proved that BUCT549 has the ability to infect a strain of *Vibrio parahaemolyticus*. A coevolution experiment determined that three strains of tolerant *V. alginolyticus* evaded phage infestation by mutating the MSHA-related membrane protein expression genes, which caused the loss of flagellum. This research on novel phage identification and the mechanism of infestation will help phages to become an integral part of the strategy for biological control agents.

## Introduction

*V. alginolyticus* is a pathogenic bacterium common in oceans and lakes, and it is prone to causing outbreaks of *Vibrio* diseases in fish, shrimp, shellfish, and other farmed animals in the aquaculture industry. Accidentally ingested *V. alginolyticus* can easily cause septicemia and other extra-intestinal infections in humans (Altekruse et al., 2000; Horseman and Surani, 2011; Mohamad et al., 2019). The frequent use of antibiotics has led to an increase of resistance strains of *V. alginolyticus*, which puts more pressure on the pathogenic bacteria in the economics and medical fields. *V. alginolyticus* has been the third most common *Vibrio* species in human disease reports for many years (Jacobs Slifka et al., 2017). In recent years, there has been an upward trend in the incidence of water pollution and food poisoning caused by *V. alginolyticus*. It worth paying more attention to *V. alginolyticus*, and it is urgent to develop alternative approaches of antibiotics to control these pathogens (Newton et al., 2012; Osunla and Okoh, 2017).

Bacteriophages, the most abundant organisms on Earth, can be found in all corners in worldwide distribution. They are gradually being developed as an alternative to antibiotics for the treatment of various bacterial infections. Phages in the ocean have an important role in carbon and energy cycling (Suttle, 2007). Compared with antibiotics, phages have many advantages, such as high specificity, obvious antibacterial effect, and simple isolation and preparation, and they are gradually being applied in the farming and medical industries. Here, we isolated a *V. alginolyticus* from farmed sick shrimp and obtained a novel *V. alginolyticus* phage designated as BUCT549 (GenBank accession no. MT735629.1) from the sewage in a nearby seafood market. Phage BUCT549 has the ability to infect both *V. alginolyticus* and *V. parahaemolyticus*. In this study, we identified its biological and genomic characteristics and screened the tolerant bacteria by coculture of phage and bacteria to promote coevolution. Furthermore, we identity that the host receptor of phage BUCT549 was an MSHA type transmembrane protein, which provides more basis for the subsequent use of the phage to control *Vibrio* hazards.

## Materials and Methods

### Isolated and Identification of Bacterial Pathogens

*V. alginolyticus* was isolated as a host from diseased shrimp in a farm in 2019 (Qingdao, Shandong, China) and cultured at 37°C using 2216E plates (2216E agar, Solarbio). The bacterial species of the host was identified by 16S universal primers (16S-F-5′-AACTGGAGGAAGGTGGGGAT-3′, and 16S-R-5′-AGGAGGTGATCCAACCGCA-3′) and Sanger sequencing.

### Isolation and Purification of BUCT549

Sewage from a seafood market in Qingdao was centrifuged at 10,000 × g for 10 min, and the supernatant was filtered through a 0.22-μm syringe filter, 50 μL of filtrate was mixed with 5 mL of log phase (OD_600_≈0.5) *V. alginolyticus* culture, incubated overnight at 37°C and 220 rpm. The culture solution was centrifuged at 8,000 × g for 10 min, and the supernatant filtered through a 0.22-μm filter to obtain a phage stock solution. The filtrate was applied to make double-layer plates and cultured at 37°C until translucent individual plaques appeared. A single plaque was picked and added to 5 mL of 2216E liquid medium containing 100 μL of *V. alginolyticus* inoculum and cultured at 37°C for 6 h. The phage was obtained again by centrifugation, filtration, dilution, and plating. This step of phage purification was repeated three times.

### High-Throughput Genome Sequencing of Phage BUCT549

Phage genomic DNA was extracted using a modified phenol-chloroform extraction protocol (Li et al., 2019). A 2 × 300 nt paired-end DNA library was prepared with the NEBNext^®^ UltraTM II DNA Library Prep Kit for Illumina following the manufacturer’s protocol^1^. Briefly, 150 ng of DNA were dissolved in deionized water to a final volume of 50 μL and disrupted to 300-bp fragments using a Bioruptor UCD-200TS ultrasound system. Then, the fragmented DNAs were end-repaired and adaptor ligated using NEBNext Ultra II End Prep Enzyme and Ligation Master Mix, respectively. Next, the adaptor-ligated DNA was selected and cleaned using EBNext Sample Purification Beads. Finally, the adaptor-ligated DNA was subjected to PCR amplification, and the PCR products were cleaned using EBNext Sample Purification Beads. High-throughput sequencing of the DNA was performed on an Illumina MiSeq instrument (San Diego, CA, United States).

### Transmission Electron Microscopy (TEM)

Phage particles were centrifuged at 13,000 × g for 2 h and then purified by sucrose density gradient centrifugation to visualize phage morphology by TEM (Chen et al., 2018). A 20-μL aliquot of phage suspension was incubated on a carbon-coated copper grid for 15 min and then dried using filter paper. The copper grid covering the phages was then stained with 2% (w/v) phosphotungstic acid (pH7.0) for 2 min. Finally, phage morphology was examined at 80 kV using a JEM-1200EX transmission electron microscope (Jeol Ltd., Tokyo, Japan).

### Multiplicity of Infection (MOI) and One-Step Growth Curves

The optimal MOI and one-step growth curves for isolated phages were determined using methods described previously (Xi et al., 2019). Briefly, phages were added to 5 mL of log-phase *V. alginolyticus* culture to achieve an MOI of 10, 1, 0.1, 0.01, 0.001, or 0.0001, and then incubated at 37°C, 220 rpm for 6 h. Culture supernatant was then filtered through a 0.22-μm filter, and the titer of the phage in the supernatant was measured using a double-layer agar plate method. Three replicates were conducted for determination. The MOI resulting in the highest phage titer was considered the optimal MOI of the phage.

The one-step growth curve of a phage reflects dynamic changes in the number of particles during phage replication. To obtain a one-step growth curve for BUCT549, phage suspension was added to 20 mL of log-phase *V. alginolyticus* culture at the optimal MOI and incubated at 37°C for 5 min. Then, the culture was centrifuged at 12,000 × g for 1 min and the supernatant discarded. The pellet was then washed twice with 2216E liquid medium and resuspended in 20 mL of 2216E liquid medium. The moment when the pellet was resuspended in medium was defined as time zero. Then, the resulting culture was transferred to a shaker and incubated at 37°C, 220 rpm for 3.5 h. Three duplicate samples (100 μL) were collected every 20 min to determine the phage titer at different time points. Three replicates were conducted for determination. The one-step growth curve was obtained by plotting phage titer against time. The burst size was calculated by dividing the plateau phage titer by the initial phage titer.

### Thermal and pH Stability

In the thermostability assay, the phage isolations were incubated at 40, 50, 60, and 70°C, and the aliquots were collected after 20, 40, and 60 min to be titered by the double-layer agar method. For the pH stability assay, samples of the isolated phage were mixed in a series of tubes containing 2216E liquid medium of different pH values [2–12, adjusted using NaOH (1 mol/L) or HCl (1 mol/L)], incubated for 1 h at 37°C, and then titered by the double-layer agar plate method.

### Tolerant Bacteria Screening

Three independent cultures of *V. alginolyticus* in logarithmic phase were used to coculture with phage BUCT549, and after observing the reclouding of the culture, the surviving bacteria were used to delineate and pick single colonies for liquid culture, and tolerance to BUCT549 was confirmed after a spot assay and DAL assay. Bacteria nucleic acids were extracted using the Bacteria Genomic DNA Kit (cwbiotech) and the DNA library was prepared with the NEBNext^®^ UltraTM II DNA Library Prep Kit for Illumina high-throughput genome sequencing.

### Bioinformatics Analysis

Sequencing data were filtered using Trimmomatic v0.36 (Bolger et al., 2014) and assembled using SPAdes v3.13.0. Bacterial drug resistance genes are transmitted through ResFinder^2^ (Bortolaia et al., 2020). Phage’s tRNAs were detected using tRANscan-SE2^3^ (Lowe and Eddy, 1997; Lowe and Chan, 2016); the virulence and pathogen genes carried by phages detected with VirulenceFinder^4^ (Clausen et al., 2018) and PathogenFinder^5^ (Cosentino et al., 2013). Rho---independent transcription terminators were projected by ARNold^6^ (Gautheret and Lambert, 2001). Open reading frames (ORFs) were predicted with RAST^7^ (Brettin et al., 2015). The ORFs were annotated using the BLASTp algorithm with the non-redundant (nr) protein database of the National Center for Biotechnology Information (NCBI)^8^. Phage homology calculations were performed using VIRIDIC^9^ (Moraru et al., 2020). Use the SNP plugin in CLC Genomics Workbench 12.0 to find tolerant bacterial mutant loci, using clustalw^10^ and ESPript^11^ (Robert and Gouet, 2014) to determine mutant site translation. Comparative genomic analysis used Easyfig 2.2.3 (Sullivan et al., 2011). To determine the taxonomy of the isolated phages, phylogenetic analysis based on the terminase large subunit was carried out using software MEGA7 with the neighbor-joining method and 1,000 bootstrap replications and the tree optimized with Evolview (Subramanian et al., 2019). Shared gene analysis was performed using OrthoMCL (Chen et al., 2006), shared gene relationships were mapped using Cytoscape 3.7.1, and phage shared gene heat maps with BUCT549 were mapped using the R package pheatmap.

## Results and Discussion

### Isolation and Identification of BUCT549 and Its Host

**FIGURE 1:**
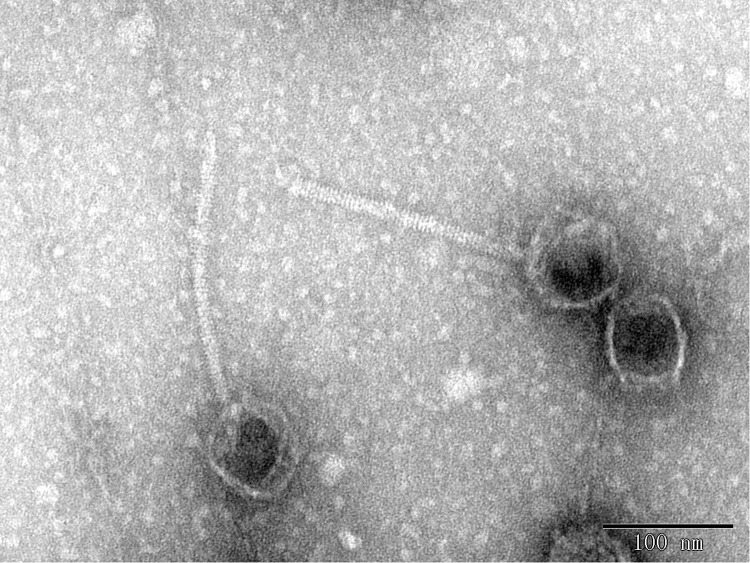
Phage morphology observed using TEM.

*V. alginolyticus* was isolated from the farmed shrimp with disease in Qingdao, China. We determined the antibiotic resistance of the bacterium from assembled NGS data by ResFinder. *V. alginolyticus* carries resistance genes for doxycycline [tet (35)], tetracycline [tet (34)] and tet [(35)], ampicillin (blaCARB-42), amoxicillin (blaCARB-42), and piperacillin (blaCARB-42), which may be the main reason for the failure to completely kill the bacteria during the culture process, thus causing diseases among shrimp. We used this bacterium as the host and collected the sewage from a nearby seafood market. Continuous purification using a double-layer agar plate finally yielded the lytic phage BUCT549, which can infect this *V. alginolyticus* strain. The results of TEM showed that phage BUCT549 was a typical phage of the family *Siphoviridae* within the *Caudovirales* order, which had an icosahedral head and a curved tail. The head diameter and tail length were approximately 186.1 ± 0.70 nm (*n* = 3) and 74.67 ± 0.41 nm (*n* = 3), respectively (Figure 1).

### Biological Characterization of BUCT549

**FIGURE 2:**
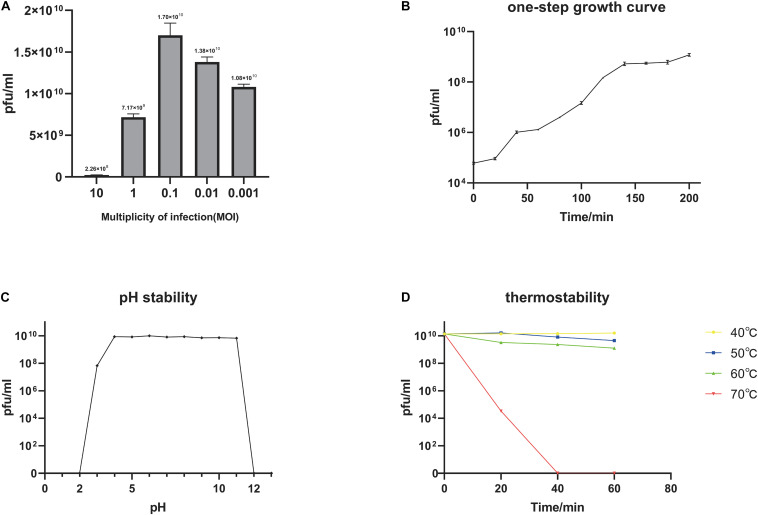
Biological characterization of the phage BUCT549. **(A)** The MOI test of BUCT549. **(B)** The one-step growth curve of phage BUCT549, and data points show phage titers measured at 20-min intervals. **(C)** pH stability of BUCT549, and data points are phage titers measured after incubation of phage at different pH for 1 h. **(D)** Thermostability curve of BUCT549, and data points are phage titers measured after incubating the phage at different temperatures for 20, 40, and 60 min, respectively. All assays were performed in triplicate.

We determined the MOI of phage BUCT549 and found that the highest viral titer of it could reach 1.7 × 10^10^ pfu/mL when the MOI was equal to 0.1 (Figure 2A). The one-step growth curve of phage BUCT549 was determined under the optimal MOI. The latent period was approximately 30–40 min, and the burst size was about 141 pfu/cell (Figure 2B). The burst size was defined as the ratio of the final number of free phage particles to the number of infected bacterial cells during the latent period.

Furthermore, we tested the stability of BUCT549, which showed that it was observably stable in lytic capacity between pH 3.0 and 11.0 by plaque counting (Figure 2C). During the temperature sensitivity assay, it was found that phage BUCT549 could be stable at 60°C for 1 h, and the activity of BUCT549 was not affected below 60°C (Figure 2D). The stability at room temperature or even at higher temperatures simplifies the conditions for phage storage or transport.

A double-layer agar plate test showed that phage BUCT549 could infect a strain of *V. parahaemolyticus* additionally. MLST typing was used to authenticate that this *V. parahaemolyticus* type is ST-772. The analysis of the NGS data of this *V. parahaemolyticus* revealed the presence of various resistance genes, such as streptomycin, sulfamethoxazole, ampicillin, and tetracycline. The ability of BUCT549 to infect resistant bacteria across species provides the possibility of its practical application, highlighting that it could be explored for phage therapies for infections of multiple drug-resistant *Vibrio* infections.

### Coevolution Identifies the Phage BUCT549 Target Proteins

**FIGURE 3:**
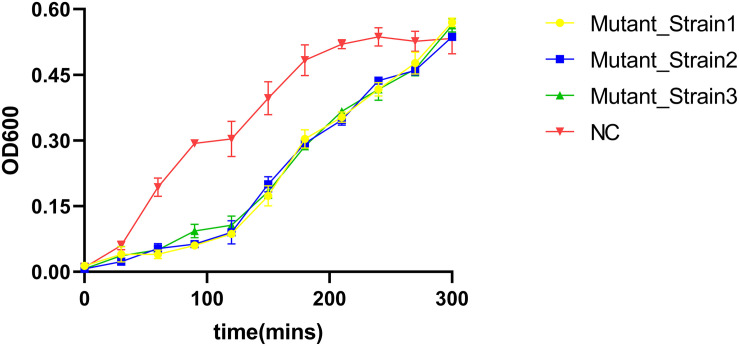
*V. alginolyticus* kill curve. *V. alginolyticus* was incubated with/without the addition of the phage, and the curve was plotted by measuring *V. alginolyticus* OD600 at 30-min intervals.

**FIGURE 4:**
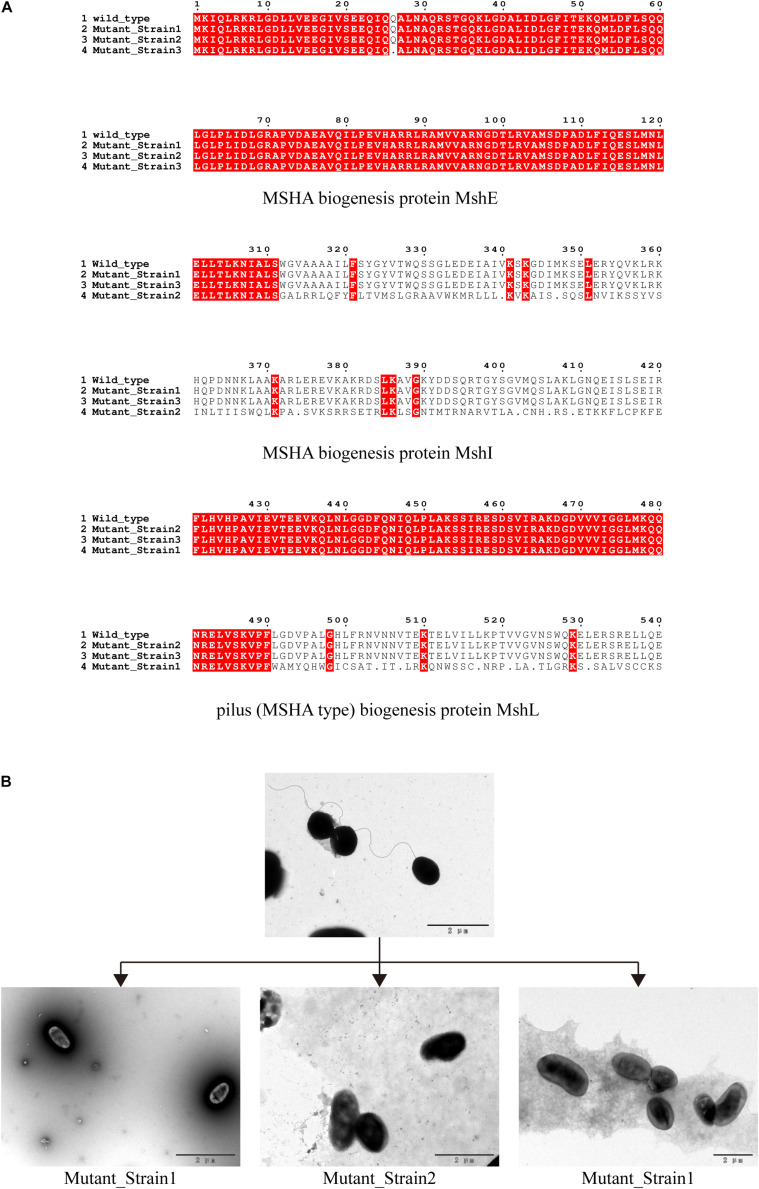
Identification of phage recognition receptors based on coevolution. **(A)** Mutant strains tolerant to phage BUCT549, MSHA-like proteins affected by 100% mutated site. Mutant_Strain3 mutates at the 26th amino acid of MshE protein to produce a termination codon. Mutant_Strain2 has a deletion at amino acid 312 of the MshI protein, producing a stop codon at amino acid 340. Mutant_Strain1 has a deletion at amino acid 491 of the MshL protein, producing a stop codon at amino acid position 504. **(B)** Electron microscopic observation of the morphology of the host *V. alginolyticus* (with flagella) and three tolerate strains (without flagella).

We determined the inhibition of *V. alginolyticus* growth by comparing the OD_600_ of *V. alginolyticus* with/without the addition of BUCT549. The *V. alginolyticus* was able to be kept at low levels for 120 min by BUCT549, but eventually it could survive with the existence of BUCT549 (Figure 3). To determine the ability that *V. alginolyticus* escapes from BUCT549, we purified the single colonies of evolved *V. alginolyticus* from three groups that cocultured with BUCT549 independently. This stable evolution of resistance was confirmed by spot and DLA tests. It was determined that the three independently screened mutant strains could resist BUCT549. The three mutant strains (named Mutant_Strain1, Mutant_Strain2, and Mutant_Strain3) were subjected to NGS to identify the reasons for their acquisition of BUCT549 resistance. By comparison with sensitive strains using single nucleotide polymorphism (SNP) analysis in CLC software, we found 100% mutated sites in each of the tolerant strains. By comparison between these complete mutation sites, we found that all three independently generated mutant strains were mutated in proteins associated with the mannose-sensitive hemagglutinin MSHA biogenesis and caused various degrees of premature termination. Mutant_Strain3 mutates at the 26th amino acid of the MshE protein to produce a termination codon. Mutant_Strain2 has a deletion at amino acid 312 of the MshI protein, producing a stop codon at amino acid 340. Mutant_Strain1 has a deletion at amino acid 491 of the MshL protein, producing a stop codon at amino acid position 504 (Figure 4A). Besides this, the tolerant bacteria observed by electron microscopy did not have flagella present (Figure 4B). MSHA is a class of proteins associated with *Vibrio* movement and involved in biofilm formation (Utada et al., 2014). It is a potential settlement factor and protective antigen, affecting bacterial adhesion (Jonson et al., 1994). Studies report that it can be the receptor for *Vibrio cholerae* phage (Jouravleva et al., 1998; Campos et al., 2010). A large number of studies focus on *Vibrio cholerae*, but so far, this is the first reported infestation that *V. alginolyticus* phages could use flagella as a recognition receptor to infect. Our study suggests an evolutionary pathway for *Vibrio* to abandon some functions by MSHA protein mutation for escape phage.

### Genomic Identification of the Phage BUCT549

**FIGURE 5:**
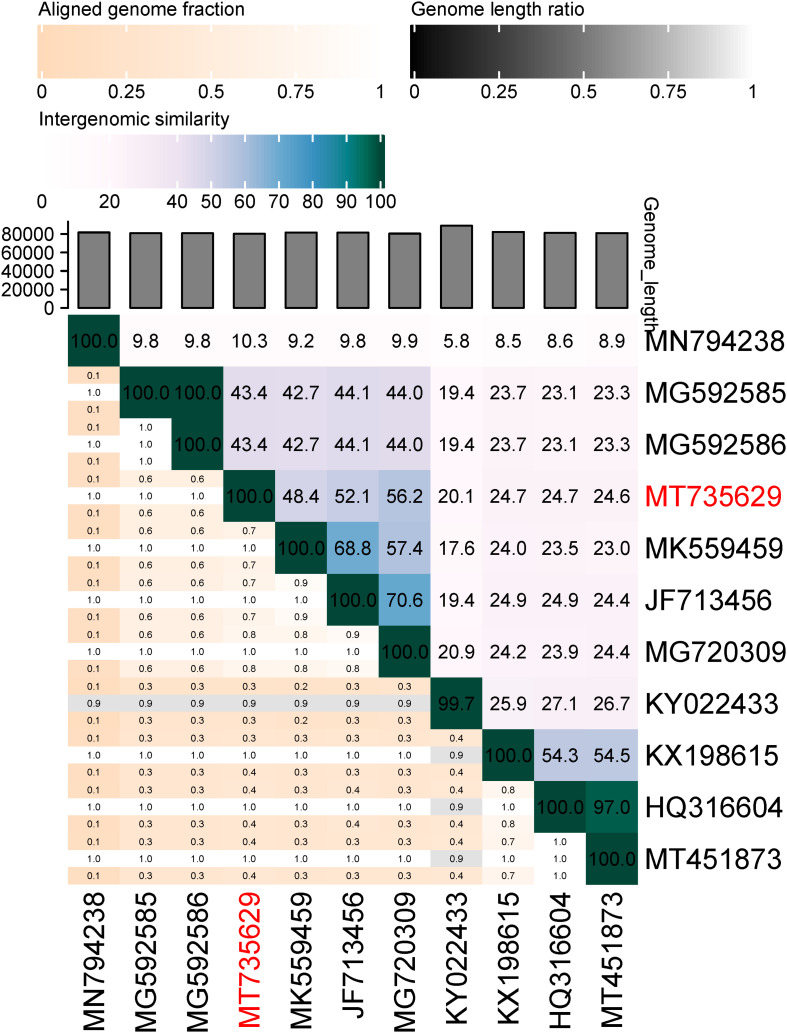
Percentage sequence similarity between phages calculated using VIDIRIC. The horizontal and vertical coordinates indicate the corresponding phage Genebank number, and the phage in this study is marked in red font.

The genome of BUCT549 was obtained using the Illumina sequencer platform, yielding 546,742 raw reads with an average length of 300 bp. By *de novo* assembly of these reads, a single contig of 80,294 bp in length and sequencing depth of 212 × was obtained with GC content of 45.54%. The genome of phage BUCT549 showed high sequence identity (76.32, 76.22, 75.27%) to the genomes of *Vibrio* phage 1 (GenBank: JF713456), *Vibrio* phage Ares1 (GenBank: MG720309) and *Vibrio* phage vB_VcaS_HC (GenBank:MK559459.1) with 37, 40, and 35% coverage, respectively. The low homology with known sequences showed that it is a novel discovered *V. alginolyticus* phage. We calculated the percentage of sequence similarity by VIRIDIC, resulting that the maximum similarity between BUCT549 and other known sequences is 56% (Figure 5), sufficient to qualify it as a new species. The International Committee on Taxonomy of Viruses (ICTV) describes a genus as a cohesive group of viruses sharing at least 60–70% nucleotide identity over the entire genome length (thresholds depending on the group of viruses) (Adriaenssens and Brister, 2017). Therefore, we are proposing to create a new genus in the *Siphoviridae* family to phage BUCT549.

**FIGURE 6:**
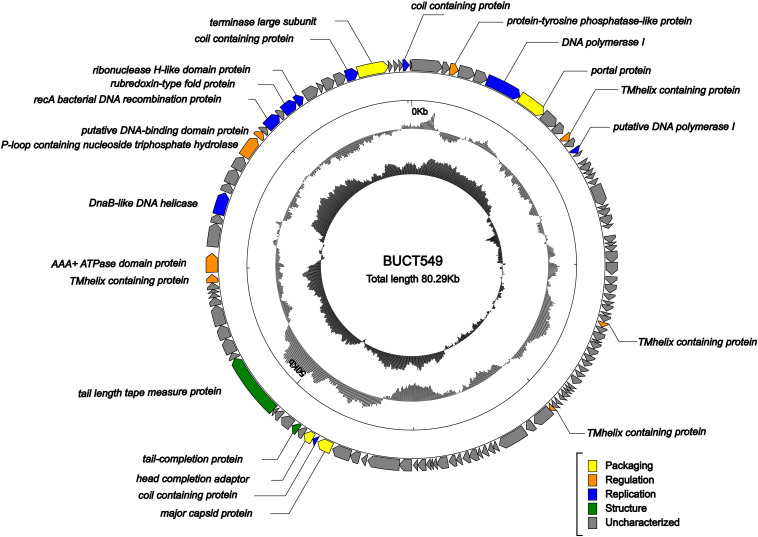
Genome map of BUCT549. The outermost circle represents ORFs encoded in the genome with different colors representing different functions (clockwise arrow indicates the forward-reading frame, counterclockwise arrow indicates the reverse reading frame); the dark circles in the middle represent the GC content (outward indicates greater than the average GC content compared with the whole genome, and inward indicates the opposite); the innermost circle represents the GC skew (G–C/G + C. Outward indicates > 0 and inward indicates < 0).

**FIGURE 7:**
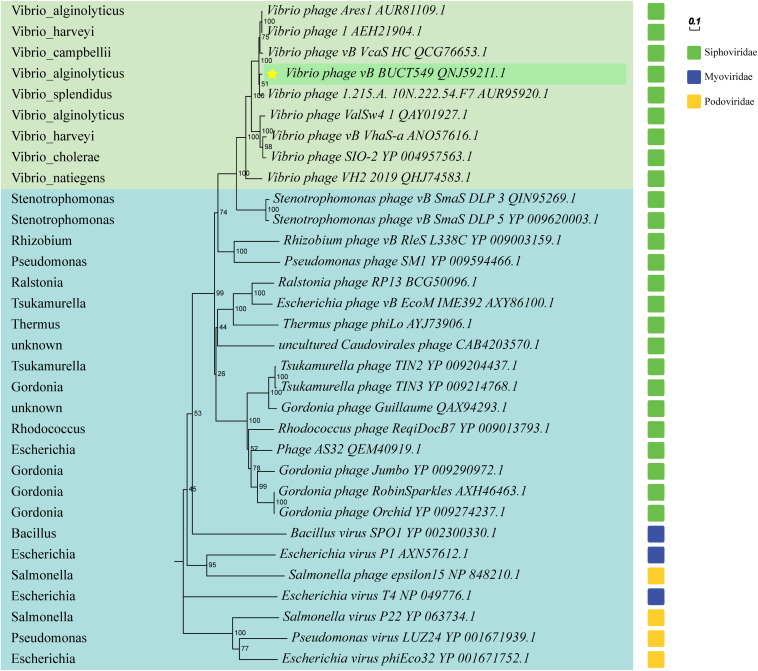
Phylogenetic trees based on terminase large subunit, and partial phages of the *Myoviridae* and *Podoviridae* are selected as outgroups. *Vibrio* phages are marked in light green, and other host phages are marked in light blue. Hosts for all phages are shown on the left of the phylogenetic trees.

ORF analysis of the phage using RAST determined that BUCT549 contains 119 predicted ORFs (Figure 6). In addition, we found 61 transcriptional terminators and no tRNA in BUCT549, indicating that BUCT549 is dependent on the host’s replicative-translational machinery to complete its life activities. Phage BUCT549 did not carry any virulence or pathogenicity genes determined by VirulenceFinder and PathogenFinder, suggesting its potential application as an antibacterial and therapeutic agent. Using Blastp to annotate all ORFs, phage BUCT549 contains a large number of proteins of unknown function. Interestingly, we found that a lot of protein (81/119) annotation information for BUCT549 has similarity to *Vibrio* phage 1, which uses *Vibrio harveyi* as the host reported in 2012 (Khemayan et al., 2012). Using multiple-sequence alignment with *Vibrio* phage 1 and *Vibrio* phage Ares1, we found that BUCT549 was homologous to phage strains only in a relatively concentrated portion of the genome, and the homology coverage is less than 40% (Figure 7). A total of 18 proteins were successfully identified by protein profile, including portal protein, major capsid protein, head completion adaptor, tail length tape measure protein and other major structural proteins (Supplementary Table 1).

**FIGURE 8:**
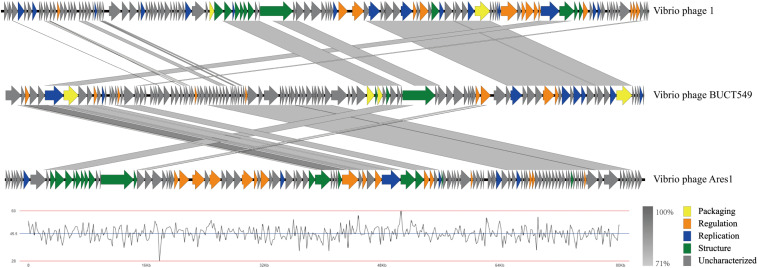
Multiple-sequence alignment of phage genomes. The whole genomes of *Vibrio* phage BUCT549, *Vibrio* phage 1, and *Vibrio* phage Ares 1, compared using Easyfig. The gray shading indicates sequence similarities between the genomes. The lower half of the graph indicates the GC content of phage BUCT549.

The phylogenetic tree was constructed using terminase large subunit of the phage, and some phages from the family *Myoviridae* and *Podoviridae* were selected as outgroups (Figure 8). BUCT549 was clustered with *Siphoviridae* phages, which is consistent with electron microscopy results. All the phages close to BUCT549 in the phylogenetic tree infect *Vibrio* bacteria, but they are quite different from each other (*Vibrio* phages are shown in the light green background and hosts are marked in the left). Phages under the same branch are able to infect *Vibrio harveyi* (*Vibrio* phage1 and *Vibrio* phage vb_VhaS-a), *Vibrio campbellii* (*Vibrio* phage vb_VcaS_HC), *Vibrio splendidus* (*Vibrio* phage 1.215.A. 10N.222.54.F7), *Vibrio cholerae* (*Vibrio* phage SIO-2), and *Vibrio natiegens* (*Vibrio* phage VH2_2019). The ability of *Vibrio* phage to infect across species has often been reported (Chen et al., 2020; Misol et al., 2020), *Vibrio* phages have great potential to treat mixed *Vibrio* contamination.

### Core Gene Identification of Phage BUCT549

**FIGURE 9:**
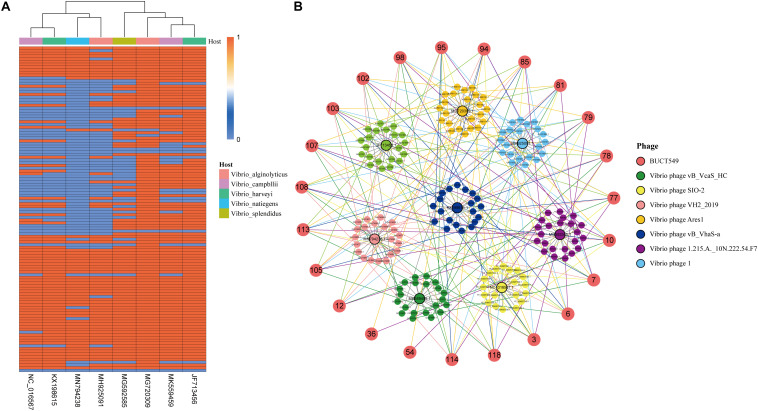
Analysis of genes shared by BUCT549 with other *Vibrio* phages. **(A)** The heat map shows the different *Vibrio* phage genes compare to BUCT549 with red representing phage sharing the gene with BUCT549 and blue representing not sharing the gene. Phage host species are colored above the heat map. **(B)** Relationship between the annotated genes of BUCT549 and other phage genes, the connecting lines represent the gene shared by phages.

BUCT549 and another eight vibrio phages closest to BUCT549 were selected for core gene identification. Homology analysis and clustering were carried out with all annotated proteins, and a total of 159 clusters were divided into nine phages. The number of genes shared by different phages is independent of the species of the host. BUCT549 shares 112 genes with *V. alginolyticus* phage Ares1 but shares 64 genes with another *V. alginolyticus* phage ValSw4_1. The number of genes that BUCT549 shares with two *V. harveyi* phages or two *Vibrio campbllii* phages is also different. BUCT549 shares 105 genes with *V. harveyi* phage 1 and 72 genes with *V. harveyi* phage vB_VhaS-a, and it shares 98 genes with *V. campbllii* phage vB_VcaS_HC and 72 genes with *V. campbllii* phage SIO-2 (Figure 9A). The 23 annotated genes are linked to other phages using different colored lines (Figure 9B). Nineteen genes, including DNA polymerase (ORF6), portal protein (ORF7), major capsid protein (ORF77), tail proteins (ORF81 and ORF85) and terminase large subunit (ORF114) present in all *Vibrio* phages, demonstrate the feasibility of using such conserved proteins coded by core genes to determine evolutionary relationships. The amount of TMhelix containing protein in *Vibrio* phages is different, two TMhelix containing proteins are shared in nine phages and two TMhelix containing proteins are only present in three phages. The interaction between TMhelix protein plays a key role in the structure and function of membrane proteins (Zhang and Lazaridis, 2009), and the TMhelix proteins in the phage may be involved in the regulation of host bacterial membrane proteins.

## Conclusion

In the present study, we identified a novel phage strain in the family *Siphoviridae*, which uses the *V. alginolyticus* as a host. There is no virulence and pathogenicity genes in its genome, and it has the ability to be used as a potential antimicrobial agent. Simultaneously, BUCT549 grew over a wide pH (3.0–11.0) and temperature (up to 60°C) range, which showed it can be used as a potential antibacterial agent in aquatic products or the medical industry. Biological characteristics of phage BUCT549 and the analysis of genomic data find that the phage could infect the host with MSHA-related proteins in the host flagellum as the receptor and complete the life process. Flagella are widespread in *Vibrio*, and deeper mining of this kind of receptors could help broaden the range of hosts for phage application. We also observed that bacteria are able to evolve by shedding flagella to escape phage recognition. Meanwhile, BUCT549 was able to infect a strain of *V. parahaemolyticus*, showing the broad spectrum of *Vibrio* bacteriophages. The number of genes shared by BUCT549 with other *Vibrio* phages is independent of the host species. The core genes, such as DNA polymerase, terminase large subunit, and major capsid protein, are widespread in phages, but the number of regulation proteins are different in phages, suggesting that phages differ in their ability to interact with their hosts. In conclusion, *Vibrio* phages have great potential in production and medical fields, and a detailed elucidation of phage life processes will help the rapid application of phages.

## Data Availability Statement

The sequence of the phage BUCT549 used for the study is available in Genebank under the accession number MT735629.1.

## Author Contributions

YT and TS conceived and designed the study. JL, FT, and YH performed the experiments, analyzed the data, and prepared the initial draft of the manuscript. WL and YL contributed in sequencing work. FZ, HR, and QP provided the bacteria and phage samples. All authors checked and reviewed the manuscript.

## Conflict of Interest

FZ, HR, and QP are from Qingdao Phagepharm Bio-tech Co., Ltd. The remaining authors declare that the research was conducted in the absence of any commercial or financial relationships that could be construed as a potential conflict of interest.

## Footnotes

1 https://international.neb.com/-/media/nebus/files/manuals/manuale6177-e7805.pdf

2 https://cge.cbs.dtu.dk/services/ResFinder

3 http://lowelab.ucsc.edu/cgi-bin/tRNAscan-SE2.cgi

4 https://cge.cbs.dtu.dk/services/VirulenceFinder

5 https://cge.cbs.dtu.dk/services/PathogenFinder

6 http://rssf.i2bc.paris-saclay.fr/toolbox/arnold

7 https://rast.nmpdr.org/rast.cgi

8 https://www.ncbi.nlm.nih.gov

9 http://rhea.icbm.uni-oldenburg.de/VIRIDIC/

10 https://www.genome.jp/tools-bin/clustalw

11 http://espript.ibcp.fr/ESPript/cgi-bin/ESPript.cgi
