# Supplementary material for: Characterization and Genomic Analysis of BUCT549, a Novel Bacteriophage Infecting Vibrio alginolyticus With Flagella as Receptor
Source: Front Microbiol. 2021 Jun 17;12:668319. doi: 10.3389/fmicb.2021.668319 (PMC8245777; doi:10.3389/fmicb.2021.668319)
Supplement: Supplementary file 1 [file Table_1.pdf]

Supplementary Material

1 Supplementary Table 1

ORF analysis of the BUCT549 genome

ORF	Top BLAST hit [a]	Start codon	Start	stop	strand	% identity [b] (aa)	E value [c]	Protein hited by Mass Spectrometry
1	VHS1097 protein [Vibrio phage 1]	GCC	3	1964	+	396/653(61%)	0	
2	VHS1098 protein [Vibrio phage 1]	ATG	1968	2447	+	52/130(40%)	1.00E-18	
3	protein-tyrosine phosphatase-like protein [Vibrio phage 1.215.A._10N.222.54.F7]	ATG	2450	3010	+	90/165(55%)	8.00E-51	
4	VHS1100 protein [Vibrio phage 1]	ATG	3035	4075	+	166/346(48%)	3.00E-112	
5	hypothetical protein Ares1_0058 [Vibrio phage Ares1]	ATG	4088	4927	+	159/278(57%)	7.00E-110	
6	DNA polymerase I [Vibrio phage Ares1]	ATG	4939	7296	+	695/785(89%)	0	
7	portal protein [Vibrio phage Ares1]	ATG	7299	9164	+	484/617(78%)	0	+
8	VHS1106 protein [Vibrio phage 1]	ATG	9168	10250	+	138/163(85%)	2.00E-96	
9	VHS1107 protein [Vibrio phage 1]	ATG	10257	10907	+	195/216(90%)	2.00E-147	

10	TMhelix containing protein [Vibrio phage 1.215.A._10N.222.54.F7]	ATG	11096	11521	+	89/141(63%)	6.00E-56	+
11	VHS1109 protein [Vibrio phage 1]	ATG	11534	11989	+	99/150(66%)	6.00E-70	
12	putative DNA polymerase I [Vibrio phage Ares1]	ATG	12118	12492	+	76/121(63%)	7.00E-51	
13	VHS1111 protein [Vibrio phage 1]	ATG	12495	12686	+	45/61(74%)	6.00E-26	
14	hypothetical protein Ares1_0068 [Vibrio phage Ares1]	ATG	13073	13336	+	28/46(61%)	2.00E-09	
15	VHS1114 protein [Vibrio phage 1]	ATG	13338	13790	+	42/60(70%)	1.00E-22	
16	VHS1115 protein [Vibrio phage 1]	ATG	13794	14081	+	50/94(53%)	3.00E-29	
17	VHS1116 protein [Vibrio phage 1]	ATG	14086	14391	+	59/100(59%)	5.00E-33	
18	VHS1117 protein [Vibrio phage 1]	ATG	14477	14785	+	70/101(69%)	2.00E-46	
19	VHS1118 protein [Vibrio phage 1]	ATG	14845	16161	+	139/448(31%)	2.00E-55	
20	VHS1120 protein [Vibrio phage 1]	ATG	16229	16468	+	38/71(54%)	5.00E-17	
21	VHS1121 protein [Vibrio phage 1]	GTG	16455	16895	+	100/146(68%)	3.00E-69	
22	VHS1122 protein [Vibrio phage 1]	ATG	16901	17458	+	112/179(63%)	3.00E-75	
23	not hits	ATG	17477	17719	+			

24	VHS1001 protein [Vibrio phage 1]	ATG	18191	18634	+	55/146(38%)	4.00E-24	
25	VHS1002 protein [Vibrio phage 1]	ATG	18619	18831	+	33/56(59%)	5.00E-15	
26	VHS1003 protein [Vibrio phage 1]	ATG	18834	19178	+	50/132(38%)	9.00E-14	
27	VHS1004 protein [Vibrio phage 1]	ATG	19183	19902	+	125/232(54%)	8.00E-72	
28	VHS1005 protein [Vibrio phage 1]	ATG	19889	20677	+	139/259(54%)	3.00E-94	
29	VHS1007 protein [Vibrio phage 1]	ATG	20806	21294	+	42/142(30%)	3.00E-10	
30	VHS1008 protein [Vibrio phage 1]	ATG	21319	21864	+	93/177(53%)	6.00E-57	
31	VHS1010 protein [Vibrio phage 1]	GTG	21935	22321	+	58/107(54%)	2.00E-32	
32	VHS1011 protein [Vibrio phage 1]	ATG	22402	22644	+	58/80(73%)	3.00E-35	
33	VHS1012 protein [Vibrio phage 1]	GTG	22650	23048	+	83/132(63%)	4.00E-61	
34	VHS1009 protein [Vibrio phage 1]	ATG	23134	23328	+	39/63(62%)	1.00E-17	
35	VHS1013 protein [Vibrio phage 1]	ATG	23345	23731	+	73/125(58%)	5.00E-46	
36	TMhelix containing protein [Vibrio phage 1.215.A._10N.222.54.F7]	ATG	23782	24105	+	36/109(33%)	3.00E-08	
37	VHS1015 protein [Vibrio phage 1]	ATG	24124	24495	+	57/123(46%)	4.00E-28	

38	not hits	ATG	24496	25017	+			
39	not hits	ATG	25014	25160	+			
40	VHS1017 protein [Vibrio phage 1]	ATG	25163	25570	+	92/135(68%)	8.00E-63	
41	hypothetical protein [Vibrio phage vB_VhaS-a]	TTG	25604	26056	+	59/112(53%)	3.00E-35	
42	VHS1019 protein [Vibrio phage 1]	ATG	26078	26476	+	36/110(33%)	2.00E-10	
43	VHS1020 protein [Vibrio phage 1]	ATG	26473	26832	+	45/111(41%)	3.00E-10	
44	VHS1021 protein [Vibrio phage 1]	ATG	26924	27265	+	76/112(68%)	4.00E-45	
45	VHS1022 protein [Vibrio phage 1]	ATG	27324	27659	+	79/111(71%)	8.00E-47	
46	VHS1023 protein [Vibrio phage 1]	ATG	27714	28163	+	46/143(32%)	8.00E-16	
47	VHS1025 protein [Vibrio phage 1]	ATG	28307	28510	+	48/65(74%)	8.00E-26	
48	VHS1028 protein [Vibrio phage 1]	ATG	28514	28720	+	42/61(69%)	2.00E-22	
49	VHS1029 protein [Vibrio phage 1]	ATG	28720	28863	+	45/47(96%)	2.00E-25	
50	VHS1026 protein [Vibrio phage 1]	ATG	28908	29219	+	52/103(50%)	1.00E-23	
51	VHS1027 protein [Vibrio phage 1]	ATG	29222	29458	+	58/78(74%)	6.00E-39	

52	VHS1030 protein [Vibrio phage 1]	ATG	29599	29802	+	56/66(85%)	5.00E-32	
53	hypothetical protein Ares1_0108 [Vibrio phage Ares1]	ATG	29864	30136	+	46/87(53%)	2.00E-25	
54	TMhelix containing protein [Vibrio phage 1.215.A._10N.222.54.F7]	ATG	30133	30399	+	34/80(43%)	7.00E-13	
55	VHS1031 protein [Vibrio phage 1]	ATG	30399	31712	+	92/335(27%)	3.00E-20	
56	hypothetical protein Ares1_0111 [Vibrio phage Ares1]	ATG	31817	32365	+	153/186(82%)	6.00E-102	+
57	hypothetical protein Ares1_0112 [Vibrio phage Ares1]	ATG	32488	34362	+	419/618(68%)	0	+
58	VHS1034 protein [Vibrio phage 1]	ATG	34498	34803	+	56/98(57%)	8.00E-35	
59	hypothetical protein [Vibrio phage vB_VhaS-a]	ATG	34791	35135	+	32/68(47%)	1.00E-13	
60	VHS1036 protein [Vibrio phage 1]	ATG	35202	35564	+	68/119(57%)	1.00E-40	
61	hypothetical protein Ares1_0115 [Vibrio phage Ares1]	ATG	35618	35860	+	30/79(38%)	1.00E-05	
62	VHS1038 protein [Vibrio phage 1]	ATG	35862	36410	+	65/124(52%)	1.00E-33	
63	VHS1039 protein [Vibrio phage 1]	ATG	36407	36847	+	65/136(48%)	4.00E-38	
64	VHS1040 protein [Vibrio phage 1]	ATG	36944	37288	+	64/111(58%)	2.00E-36	
65	VHS1041 protein [Vibrio phage 1]	GTG	37298	37756	+	56/151(37%)	2.00E-28	

66	VHS1042 protein [Vibrio phage 1]	ATG	37837	38550	+	112/231(48%)	4.00E-64	
67	VHS1043 protein [Vibrio phage 1]	ATG	38537	38791	+	55/86(64%)	5.00E-30	
68	not hits	TTG	38793	38936	+			
69	VHS1044 protein [Vibrio phage 1]	ATG	38936	39226	+	38/95(40%)	1.00E-12	
70	VHS1046 protein [Vibrio phage 1]	ATG	39251	39775	+	131/174(75%)	2.00E-94	
71	VHS1047 protein [Vibrio phage 1]	ATG	39777	40052	+	59/90(66%)	4.00E-36	
72	VHS1048 protein [Vibrio phage 1]	ATG	40159	40953	+	203/264(77%)	4.00E-154	+
73	hypothetical protein Ares1_0008 [Vibrio phage Ares1]	ATG	40946	42985	+	636/649(98%)	0	+
74	VHS1050 protein [Vibrio phage 1]	ATG	43064	43402	+	42/114(37%)	3.00E-11	+
75	VHS1051 protein [Vibrio phage 1]	ATG	43537	44145	+	170/201(85%)	6.00E-125	
76	VHS1052 protein [Vibrio phage 1]	ATG	44148	45347	+	267/411(65%)	5.00E-179	
77	major capsid protein [Vibrio phage Ares1]	ATG	45435	46385	+	294/316(93%)	0	+
78	coil containing protein [Vibrio phage vB_VcaS_HC]	ATG	46475	46762	+	66/92(72%)	2.00E-40	
79	head completion adaptor [Vibrio phage 1.215.A._10N.222.54.F7]	ATG	46778	47413	+	154/212(73%)	7.00E-106	+

80	VHS1056 protein [Vibrio phage 1]	ATG	47410	47874	+	119/154(77%)	4.00E-83	
81	tail-completion protein [Vibrio phage 1.215.A._10N.222.54.F7]	ATG	47871	48338	+	107/155(69%)	4.00E-78	+
82	VHS1058 protein [Vibrio phage 1]	ATG	48393	49187	+	219/264(83%)	1.00E-154	+
83	VHS1059 protein [Vibrio phage 1]	ATG	49273	49695	+	107/140(76%)	4.00E-68	
84	VHS1060 protein [Vibrio phage 1]	GTG	49704	49895	+	23/40(58%)	4.00E-08	
85	tail length tape measure protein [Vibrio phage Ares1]	ATG	49901	54034	+	1083/1379(79%)	0	+
86	VHS1062 protein [Vibrio phage 1]	ATG	54031	54417	+	91/114(80%)	1.00E-60	+
87	VHS1063 protein [Vibrio phage 1]	ATG	54427	55410	+	168/329(51%)	5.00E-116	+
88	VHS1064 protein [Vibrio phage 1]	ATG	55424	56317	+	180/293(61%)	9.00E-129	+
89	VHS1065 protein [Vibrio phage 1]	ATG	56330	57631	+	219/432(51%)	1.00E-145	+
90	VHS1066 protein [Vibrio phage 1]	ATG	57634	58164	+	86/170(51%)	5.00E-56	
91	VHS1067 protein [Vibrio phage 1]	ATG	58157	58459	+	58/99(59%)	3.00E-35	
92	VHS1068 protein [Vibrio phage 1]	ATG	58456	58683	+	46/74(62%)	2.00E-22	
93	VHS1069 protein [Vibrio phage 1]	ATG	58673	59107	+	99/135(73%)	1.00E-70	+

94	TMhelix containing protein [Vibrio phage vB_VcaS_HC]	ATG	59110	59640	+	117/176(66%)	3.00E-81	+
95	AAA+ ATPase domain protein [Vibrio phage 1.215.A._10N.222.54.F7]	ATG	59763	60983	+	317/406(78%)	0	
96	VHS1072 protein [Vibrio phage 1]	ATG	61404	62924	+	330/505(65%)	0	
97	VHS1073 protein [Vibrio phage 1]	ATG	62921	63514	+	139/182(76%)	2.00E-99	
98	DnaB-like DNA helicase [Vibrio phage 1.215.A._10N.222.54.F7]	ATG	63499	64950	+	411/484(85%)	0	
99	VHS1075 protein [Vibrio phage 1]	ATG	65023	65613	+	92/129(71%)	6.00E-43	
100	VHS1077 protein [Vibrio phage 1]	ATG	65606	66592	+	278/323(86%)	0	
101	VHS1078 protein [Vibrio phage 1]	ATG	66605	67522	+	242/309(78%)	9.00E-174	
102	P-loop containing nucleoside triphosphate hydrolase [Vibrio phage 1.215.A._10N.222.54.F7]	ATG	67571	68998	+	391/474(82%)	0	
103	putative DNA-binding domain protein [Vibrio phage vB_VcaS_HC]	ATG	69094	69546	+	101/150(67%)	4.00E-71	
104	VHS1081 protein [Vibrio phage 1]	ATG	69552	69935	+	88/127(69%)	6.00E-63	
105	recA bacterial DNA recombination protein [Vibrio phage 1.215.A._10N.222.54.F7]	ATG	69949	71028	+	326/359(91%)	0	
106	VHS1083 protein [Vibrio phage 1]	ATG	71006	71422	+	112/135(83%)	7.00E-77	

107	rubredoxin-type fold protein [Vibrio phage 1.215.A._10N.222.54.F7]	ATG	71412	72407	+	275/330(83%)	0	
108	ribonuclease H-like domain protein [Vibrio phage 1.215.A._10N.222.54.F7]	ATG	72407	72970	+	145/186(78%)	1.00E-104	
109	VHS1086 protein [Vibrio phage 1]	ATG	73083	74048	+	261/306(85%)	0	
110	VHS1087 protein [Vibrio phage 1]	ATG	74114	74434	+	50/90(56%)	2.00E-24	
111	VHS1088 protein [Vibrio phage 1]	ATG	74462	75211	+	201/246(82%)	5.00E-152	
112	VHS1089 protein [Vibrio phage 1]	ATG	75271	76002	+	157/229(69%)	3.00E-117	
113	coil containing protein [Vibrio phage vB_VcaS_HC]	ATG	76014	76820	+	210/268(78%)	2.00E-152	
114	terminase large subunit [Vibrio phage vB_VcaS_HC]	ATG	76810	78807	+	581/655(89%)	0	
115	VHS1092 protein [Vibrio phage 1]	ATG	78813	79055	+	50/80(63%)	2.00E-30	
116	hypothetical protein Ares1_0051 [Vibrio phage Ares1]	ATG	79157	79429	+	74/89(83%)	2.00E-46	
117	VHS1094 protein [Vibrio phage 1]	ATG	79474	79740	+	57/88(65%)	3.00E-33	
118	coil containing protein [Vibrio phage vB_VcaS_HC]	ATG	79730	80149	+	90/123(73%)	9.00E-58	
119	VHS1097 protein [Vibrio phage 1]	GTG	80183	80293	+	25/31(81%)	6.00E-07	

[a] the most closely related protein and its organism. ‘‘No hits’’ indicates no significant hits detected for a particular amino acid sequence

[b] percent identity for top hits in BLASTP searches. Numbers in parentheses provide length of each alignment

[c] the probability of obtaining a match by chance as determined by BLASTP analysis
